# Supplementary material for: Learning curve and surgical outcome of robotic assisted colorectal surgery with ERAS program
Source: Sci Rep. 2022 Nov 29;12:20566. doi: 10.1038/s41598-022-24665-w (PMC9709162; doi:10.1038/s41598-022-24665-w)
Supplement: Supplementary file 2 — Supplementary Information 2. [file 41598_2022_24665_MOESM2_ESM.zip › RAL Raw data-Ñ[▒K/089/HS_ñw╝╨░O▒Kñσ.pdf]

正式摘要(電子簽章：000000218421842)<br>W76 / W76 ☐ 英文標題(注意:無英文姓名-請補病人基本檔!)	☐【讀取】憑證	

臺中榮民總醫院
出院病歷摘要

轉入號 : 轉出號 :

[redacted]

**(8)**流水編號	
**(9)**入院日期	110/05/31 (2021)
**(10)**轉科日期	
**(11)**出院日期	110/06/07 (2021) 住院天數計 7 日

診斷

- 入院 :s colon tumor, 35cm fav, pending obstruction
- 出院 :S-colon ca, 35cm FAV, s/p Robot Assisted anterior resection on 2021/6/3

主訴

- colon cancer admitted for surgery

病史

- The 75-year-old female had underlying disease of DM, HT.
  She was admitted due to incidentally found s colon tumor, 35cm fav, pending obstruction. She also complained tiredness during last week. Therefore, this time, under the impression of colon cancer, she was admitted for Robot Assisted Colectomy(L)

體檢發現

- 2021/05/30 住院前篩檢
  - General appearance :
    - easy looking , conscious clear , without cardiopulmonary distress
    - Vital signs自動帶入date 體溫(BT) 脈搏(P) 呼吸(R) 血壓(BP) 身高(BH) 體重(BW) BMI
      20210531 36.4℃ 100次/分 19次/分 127/64mmHg 150.4cm 58.2kg 25.7
    - Vital signs: B.P. 153/95 mmHg , P.R. 71 /min , R.R. 18 /min , B.T. 36.5 ℃
  - HEENT :
    - Eyes: no anemic conjunctiva, no icteric sclera, normal EOM
    - ENT: no otorrhea, no rhinorrhea, no erythematous mucosa, no injected tonsil, no deformity
  - Neck: supple, freely movable, trachea midline,no deformity, no engorged jugular vein , no palpable thyroid or lymph node enlargement
  - Chest:
    - Inspection: Grossly normal, symmetric and fully expansion, no accessory muscle used, no operation scar, no spider angiomata, no gynecomastia
    - Palpation: no palpable mass or nodule, no subcutaneous emphysema, no tenderness
    - Ausculation: clear breathing sound
  - Heart:
    - Percussion: normal heart size
    - Ausculation: regular heart beat, normal S1, S2, no gallop, no murmur
  - Abdomen:
    - Inspection: in
      - flat contour, symetrically, no scar, no stria, no dilated veins, no rashes
    - Ausculation: normoactive bowel sound, no abdominal bruits
    - palpation: soft, no muscle guarding or rigidity, no local tenderness, no rebounding pain, no palpable mass, no palpable pulsation
    - Percussion: no shifting dulllness, no hepatomegaly, no splenomagaly
  -

Peripheral pulsation :	CA	BA	RA	FA	PA	PTA	DPA

Right side :	++	++	++	++	++	++	++/+++
Left side :	++	++	++	++	++	++	++/+++

  - Genito-anus: no tenderness or palpable mass lesion or rectal shelf, normal external genitalia, normal anal tone, brown stool

CRS CONDITION

  -
    - Lung

手術日期及方法

- 手術日期及方法20210603 Robot Assisted Colectomy(L)

住院治療經過

- After admission, pre op survey was OK and nutrition support was given. The operation was done on 06/03 smoothly and hs etolerated it well. She could stand oral feeding gradually. Under relatively stable condtiions, she will be discharged.

合併症

一般檢查

- 累積報告 :

DATE	TIME	ALB	TP	BIL.T	BIL.D	ALKP	AST	ALT	LDH	AMY	LIPASE	NH3	Ferritin
1100531	1758	3.8		0.3		64		9					
1100603	1339			0.46				8					

DATE	TIME	WBC	RBC	HGB	HCT	MCV	PLT	BLST	NEUT	BAND	SEG	LYM	MON	EOS	BAS
1100531	1757	10130		6.0	21.5		306		77.7			13.8	6.8	1.4	0.3
1100601	1230			6.8											
1100602	1042			9.1											
1100603	0600			10.3	35.1										
1100603	1339	13320		9.7	31.1		252		95.8			2.9	1.2	0.0	0.1

DATE	TIME	NA	K	CL	BUN	CREAT	eGFR	CA	Mg	P	CRP	ESR	PCT	D-Dimer	LACTATE
1100531	1758	139	4.1		19	0.91	64.05	8.8	2.2	3.4					
1100603	1339	138	3.6		13	0.78	76.52	8.6							

DATE	TIME	GLUCOSE	HbA1C	CHOL	TG	HDL	LDL	KETONE	CK	CKMB	TRO-T	PT-P	APTT-P	NT-pro BNP
1100531	1758	141		119	122							10.2	23.1	

特殊檢查

病理報告

其它

出院時情況

- 3.改門診治療

出院指示

藥名	劑量	劑量單位	用法	頻次	數量	數量單位	現況	天數	單位
Jardiance tab 10mg	1	TAB	PO	QD	14	TAB	TH	14	天
5mg Bisoprolol tab	0.5	TAB	PO	QN	7	TAB	TH	14	天
Oph gel Vidisic		TUB	OU	TID	0	TUB	TH	1	瓶
Fluitran tab 2mg	1	TAB	PO	QD	14	TAB	TH	14	天
Mag. Oxide tab	2	TAB	PO	TID	84	TAB	TH	14	天
Flurbiprofen 100mg	1	TAB	PO	BID	28	TAB	TH	14	天
Trazodone tab 50mg	0.5	TAB	PO	HS	7	TAB	TH	14	天
Ultracet tab	1	TAB	PO	Q6H	56	TAB	TH	14	天
Stilnox tab 10mg	1	TAB	PO	HS	14	TAB	TH	14	天
2/500 Amaryl M tab	1	TAB	PO	BIDAC	28	TAB	TH	14	天
5/20mg Caduet tab	1	TAB	PO	HS	14	TAB	TH	14	天
Dexlansoprazole cap 60mg	1	CAP	PO	QD	14	CAP	TH	14	天
Fylin tab 400mg	1	TAB	PO	BID	28	TAB	TH	14	天

出院計劃

主治醫師 2062F 蔣鋒帆 / 住院醫師 2309E 古朋育

正式摘要(電子簽章：000000218421842)

W76 / W76

doctorRank:B
